# A Neural Machine Translation Model for Arabic Dialects That Utilises Multitask Learning (MTL)

**DOI:** 10.1155/2018/7534712

**Published:** 2018-12-10

**Authors:** Laith H. Baniata, Seyoung Park, Seong-Bae Park

**Affiliations:** ^1^School of Computer Science and Engineering, Kyungpook National University, Daegu 41566, Republic of Korea; ^2^Department of Computer Science and Engineering, Kyung Hee University, Yongin-si 17104, Republic of Korea

## Abstract

In this research article, we study the problem of employing a neural machine translation model to translate Arabic dialects to modern standard Arabic. The proposed solution of the neural machine translation model is prompted by the recurrent neural network-based encoder-decoder neural machine translation model that has been proposed recently, which generalizes machine translation as sequence learning problems. We propose the development of a multiytask learning (MTL) model which shares one decoder among language pairs, and every source language has a separate encoder. The proposed model can be applied to limited volumes of data as well as extensive amounts of data. Experiments carried out have shown that the proposed MTL model can ensure a higher quality of translation when compared to the individually learned model.

## 1. Introduction

Machine translation is an intricate process which deals with semantic, syntactic, morphological, and additional varieties of grammatical complexities, and simultaneously with multiple languages. The problem is further complicated in instances where the source language and the target language have a wide array of linguistic dissimilarities. For example, in the case of Arabic dialects which differ from the target language, such as Modern Standard Arabic at the phonological, syntactic, morphological, and lexical levels [1]. Furthermore, Arabic dialects have morphological differences; complex or compounded words are converted into simpler subunits in order to adjust the morphological symmetry [2]. Recently, the neural machine translation model has successfully obtained remarkable results in terms of translation quality. When compared to statistical machine translation methods, neural machine translation models optimize the quality and the performance of the translation by generalizing machine translation as sequence problems. On the basis of neural machine translation approaches, long-range dependencies and lexical sparsity problems in statistical machine translation can be solved through a neural network such as long short-term memory (LSTM). It can provide ideal lexical generalization and optimum long-term sequence memorization techniques.

The Arabic Language is an example of diglossia, which is defined in [3] “A relatively stable language situation in which, in addition to the primary dialects of the language, there is a very divergent highly codified superposed variety, the vehicle of a large and respected body of written literature which is learned largely by formal education and is used for most written and formal spoken purposes, but is not used by any sector of the community for ordinary conversation.” This grammatical phenomenon is found in all Arab countries. The Arabic language in its present form is in fact a collection of different varieties: Modern Standard Arabic (MSA) which represents the high register of the language, prevalent across the region. This register is commonly used in educated circles and in formal settings and has a standard orthography. Arabic dialects (AD), which are also referred to as vernaculars, are the prevalent spoken varieties of modern standard form of Arabic. Arabic vernaculars have been influenced over time by many factors, for example, those correlated with differences in cultures such as the influence of European languages and the influence of ancient local idioms. These varieties routinely appear in social media platforms, like Facebook and Twitter.

The quality of the translation deteriorates when the volume of the training data for minor languages reduces. One of the challenges encountered during the development of AD-to-MSA neural machine translation systems is the lack of available data for training. Arabic dialects are counted among languages that have fewer such available resources, and limited or no access is available to these data. Many Romance languages are official languages of specific regions with prescribed standards, naturally occurring in parallel corpora like the European Parliament [4]. AD have no official status and were rarely written until the advent of social networks and forums. The recent release of parallel multidialect corpora such as MPCA [5] has allowed for the conduction of low-resource MT experiments [6]. Another problem in developing dialectal Arabic neural machine translation systems is the lack of the standardised orthographies for all Arabic dialects and their numerous subvarieties. These include morphological differences which are clearly evident in the use of clitics and affixes that do not exist otherwise in Modern Standard Arabic.

The plausible techniques for working on Arabic dialects are still under formulation, and no previous research work has been found on applying neural machine translation on Arabic dialects. To improve the overall performance and the quality of the neural machine translation, we propose MTL in order to translate Arabic dialects to the modern standard form of Arabic. The research carried out reveals that the selected approach is successful and presents additional knowledge for the construction of the end-to-end neural machine translation model for Arabic dialects. Through the provision of combined training for several natural language tasks in one model, we can leverage the knowledge gained and enhance the performance of the translation task for all Arabic dialects.

## 2. Challenges of Arabic Dialects

Arabic dialects share many difficulties with Modern Standard Arabic. Arabic dialects are among the Semitic language with complicated templated derivational morphology. A majority of the verbs and nouns in colloquial Arabic are derived from a collection of roots, through the process of employing templates to the roots to produce stems. The templates are said to possess knowledge that indicates the morphological characteristics of words such as their gendered forms, part-of-speech tags, and their singular and plural forms. In addition, the stem may also accept prefixes and/or suffixes to further form complex words; hence, Arabic dialects are termed as highly inflected varieties. These prefixes include determiners, coordinating conjunctions, particles, and prepositions. The suffixes include gender, associated pronouns, and singular or plural form markers. It increases the number of hidden words when attempts at testing are made. This can be found in a very large number of words and, in turn, a high level of sparseness. Arabic dialects have particularities, some examples of which are explained in this section [7]:

- The lack of standardised orthography. A substantial number of words in Arabic dialects do not follow a standard orthographic system.
- A number of words that occur in Arabic dialects do not overlap with those in MSA, due to instances of language borrowing. Some examples include words such as كافيه kAfiyh “cafe” and تاتو tAtuw “tattoo,” or coinage, such as the negative particles مش mi$ “not” and بلاش balA$ “do not.” Instances of ode switching are also very common in Arabic dialects.
- The recurring linguistic practice of merging multiple words together by concatenating and dropping letters such as in the case of the word مبيجلهاش mbyjlhA$ (he did not go to her), which is a concatenation of “mA byjy lhA$.”
- Some affixes are altered in form when compared to their MSA counterparts, such as the feminine second person pronoun ك k ⟶ كي ky, and the second person plural pronoun تم tm ⟶ تو tw.
- Some morphological patterns that do not exist in MSA occur in Arabic dialects, such as the passive pattern AitofaEal, such as اتكسر Aitokasar “it broke.”
- The introduction of new linguistic features, such as the progressive ب b meaning “is doing” and the postnegative suffix ش $, which behaves like the French “ne-pas” negation construct.
- The substitution of certain letters and the mutation of consonants. For example, in the Egyptian dialects, the interdental sound of the letter ث v is often substituted by either ت t or س s, such as in كثير kvyr “much” ⟶ كتير ktyr and the glottal stop is reduced to a glide, in جائز jA}iz “possible” ⟶ جايز jAyiz. The occurrence of such features is studied in detail under the category of Phonology under lenition, which includes the softening of a consonant, or fortition, and the hardening of a consonant.
- The occurrence of vowel elongation, such as راجل rAjil “man” from رجل rajul, and vowel shortening, such as ديما dayomA “always” from دايما dAyomA.
- The use of the masculine plural or the singular noun forms, instead of the dual form or the feminine plural, dropping some articles and prepositions in some syntactic constructions, and the use of only one form of the noun and verb suffixes such as ين yn instead of ون wn and وا wA instead of ون wn, respectively.
- In addition to the above, there are the features prevalent in informal texts, such as the use of emoticons and the repetition of characters for emphasis, e.g., ادعووووووولى AdEwwwwwwwliy “pray for me.”

## 3. Related Work

In the natural language processing field, the Arabic dialects are likely to get some attention, particularly in the context of machine translation. Salloum and Habash [8] presented Elissa, which is a translation system constructed on the basis of rules meant for the conversion of Arabic vernaculars to the standard form of Arabic. This system works with Levantine (Jordanian, Syrian, and Palestinian), Egyptian, Iraqi, and Gulf Arabic dialects. Tachicart and Bouzoubaa [9] suggested a rule-based approach that relies on language models to translate the Moroccan dialect to MSA. This method is based on a morphological analysis through the use of the Alkhalil morphological analyzer, which was adapted for the purpose and extended with the incorporation of Moroccan dialect affixes and a bilingual dictionary (built from television productions scenarios and data collected from the web). The identification step in the translation process separates the dialect from the modern standard Arabic, further the text is analysed and segmented into annotated dialectal units. These outputs are linked with one or more MSA corresponding units through the use of the bilingual dictionary. In the generation phase, Moroccan sentences are selected and then passed to a language model to generate the modern standard Arabic sentences. Sadat et al. [10] presented a model for the translation of the Tunisian Arabic dialect to the standardised modern form of Arabic. This model is based on a bilingual lexicon which was designed for the particular context of this translation exercise. It uses a set of grammatical mapping rules with an additional step for the purpose of disambiguation which is based on a language model of Modern Standard Arabic to choose the best possible translated target phrase, and it is a word-based translation system. The model secured a BLUE score [11] of 14.32, in a test set consisting of 50 sentences from the Tunisian dialect. Furthermore, a rule-based approach was proposed by Al-Gaphari and Al-Yadoumi [12] to transform the Sanaani dialect to Modern Standard Arabic. Sanaani dialect is used in the capital city of Yemen. The system designed gave 77.32% of accuracy when it was tested on the Sanaani corpus of 9386 words.

Most of the methods mentioned above focused on rule-based methodology which applies a set of linguistic rules that allow the words to be put in different places and to have different meaning depending on context. However, rule-based machine translation (RBMT) systems have a big drawback: the construction of such systems demands a great amount of time and linguistic resources; as a result, it is very expensive. Moreover, in order to improve the quality of a RBMT, it is necessary to modify rules, which requires more linguistic knowledge. Modification of one rule cannot guarantee that the overall accuracy will be better.

On contrary, Meftouh et al. [13] presented PADIC which is a multidialect Arabic corpus that covers Modern Stranded Arabic, the Maghrebi dialects (Tunisian and Algerian), and the Levantine dialects (Syrian and Palestinian). Unlike recent work in the area, some experiments were conducted on several statistical machine translation systems that ran through all possible pairs of languages (Modern Standard Arabic and dialects). The authors investigated the importance of using the proposed language model on machine translation by employing smoothing techniques and by including them within a larger framework. They achieved satisfactory results when translating among the various dialects within Algeria, largely due to the shared vocabulary. It was remarked that the statistical machine translation performed significantly well when the translation was between the Palestinian and the Syrian dialects. This was due to the linguistic proximity of the two vernaculars; with respect to translations into Modern Standard Arabic, remarkable results were obtained with the Palestinian dialects.

Abo Bakr et al. [14] introduced a general approach to convert sentences from the Egyptian dialect into vocalized renditions of MSA sentences. In order to automatically tokenize and tag Arabic sentences, they used the statistical approach. A method based on certain rules was used for the sake of creating diacritics for target sentences in Modern Standard Arabic. The work was evaluated on a dataset of 1K of Egyptian dialect sentences (including training and test 800 and 200, resp.). For converting dialect words to MSA words, the system achieved an accuracy of 88%, whereas for producing these words into their correct order the system performed 78%. However, statistical machine translation approach presents some weaknesses. SMT requires high computational resources and cannot handle one of the syntactic issues in Arabic dialects which is the word ordering problem. Analysis of the word order involves figuring out where the subject, object, and the verb occur in the sentences. Based on this, languages could be classed as SVO (English), SOV (Hindi), and VSO (Arabic). Some languages, such as Arabic dialects allow a free word order. This means that the word order does not convey information about subject and object, but instead conveys something different possibly old and new information. These deeper differences pose challenges to SMT because as sentences get longer in length, they are no longer simple enough to contain a subject, object, and a verb but are complex constructions made up of several sentential components.

Recently, models based on multitask learning (MTL) have achieved remarkable results where a multiple learning tasks are solved at the same time, while exploiting commonalities and differences across tasks. For example, Collobert et al. [15] proposed a unified neural network design and a learning algorithm that can be used to different natural languages processing tasks such as the part-of-speech tagging, name entity recognition, chunking, and semantic role labelling. The basic multitask architecture of this model is to share some layers to define and determine common features. After the shared layers, the remaining layers are divided into various specific tasks. Rather than utilizing man-made input features optimized for each task, the model learns the internal representation on the basis of large amounts of mostly unlabelled training data. Additionally, the CNN model was used in this work.

Liu et al. [16] proposed a multitask learning architecture to learn across several tasks jointly. Based on the recurrent neural network architecture (RNN), three various methods of sharing information were used to model the text with task-specific and shared layers. The complete network is trained on all these tasks jointly. The results of the experiments on four benchmark test classification tasks show that the suggested models are able to improve the performance of the task with the help of other tasks. Niehues and Cho [17] showed that multitask learning approach is successful and introduced an additional knowledge into an end-to-end neural attentional model. By training various natural language processing (NLP) tasks jointly in one system, the model is able to leverage the shared information and enhance the performance of the individual task. The experiments are carried from German to English translation task. Part-of-speech (POS) tagging information and named entities (NE) were exploited as additional linguistic resources. The result of experiments shows that the translation quality can be increased by up to 1.5 BLUE points under the low-resource condition. The performance of POS tagger is also enhanced using the multitask learning scheme.

The proposed MTL model in this research proved to be an effective approach to improve the performance of Arabic dialect translation task with the help of other related tasks. By sharing one decoder across all tasks and using separate encoders for each source language, the proposed MTL model is able to leverage the useful information contained in multiple related tasks. Furthermore, the proposed MTL model can learn to generate the sentence in a right target language order and make the translation clearer and more fluent. No previous research work has focused on using one decoder to perform multiple translation tasks for Arabic dialects based on the multitask learning approach.

## 4. Neural Machine Translation (NMT)

**Figure 1:**
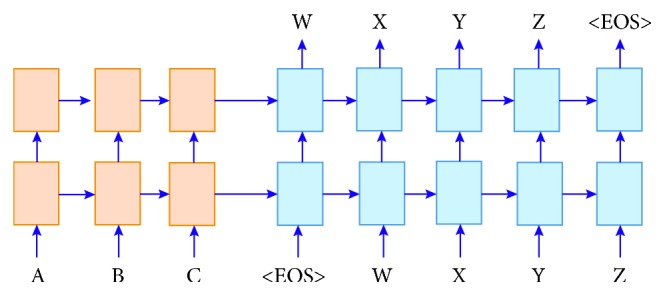
The encoder-decoder architecture for the neural machine translation (NMT). Here, the Source sentence A, B, and C is translated into a target sentence W, X, Y, and Z. At each step, an evolving real-valued vector summaries the state of the encoder (pink) and decoder (blue).

Lately, neural machine translation (NMT) has become a highly rated and preferred method and is considered to be better than the traditional statistical machine translation (SMT) models. Bentivogli et al. [18] elaborated the experimental results on the comparisons between the SMT and NMT models and provided the information that, for various cases, the results made evident through NMT outperform those obtained from the SMT models. Cho et al. [19] and Sutskever et al. [20] were able to design a powerful architecture for machine translation. In this work, we utilize a two-layer encoder-decoder system (Figure 1) with long short-term memory (LSTM) units.

In the encoder-decoder architecture which was discussed by Peyman et al. [21], two recurrent neural networks (RNNs) are trained together to maximize the conditional probability of a target sequence (candidate translation) *y* = *y*_1_,…, *y*_*m*_, given a source sentence *x* = *x*_1_,…, *x*_*n*_. Input words are sequentially processed consecutively until the end of the input string is reached. An encoder scans words and maps the input sequence into a representation with a fixed length. At each time in step*t*, an input word is taken and the hidden state is further updated. This process can be expressed in the following equation:

(1) $$\begin{array}{c} h_{t}=f\left(\;E_{x}\left[\;x_{t}\;\right],h_{t-1}\;\right) \end{array}$$

where *h*_*t*_*εR*^*d*^ the hidden state (a vector) is at the time step *t* and *f*(.) is a recurrent function such as long short-term memory (LSTM) [22] or gated recurrent unit (GRU). *f*(.) is responsible for updating the hidden state of the layer and other associated units (if there are any, such as memory units), and *E*_*x*_ ∈ *R*^|*V*_*x*_|×*d*^ is an embedding matrix for source symbols (*d* is the embedding size). The embedding matrix is a lookup table (LUT) whose cells are treated as network parameters and updated during training. The embedding (numerical vector) for the *v*th word in *v*_*x*_ (vocabulary) resides in the *v*th row of the table. In the next step, the model undertakes processing for all words in the source sequence; *h*_*n*_ is a summary of the input sequence which is referred to as the context vector (*c*). Another RNN is initialized by *c* and seeks to produce a target translation. There is one word sampled from a target vocabulary *v*_*y*_ at each step of the process. The decoder conditions the probability of picking a target word *y*_*t*_on the context vector, the last predicted target symbol, and the decoder's state. This can be expressed in the following equations:

(2) yt=gEyyt−1,St,cSt=fEyyt−1,St−1,c

where *S*_*t*_ is the decoder's hidden state. Since we compute the probability of selecting *y*_*t*_ as the target word, *g*(.) should give a value in the range [0,1]. The most common function for *g*(.) is softmax. The encoder and decoder RNNs are trained together to maximize the log probability of generating a target translation and are given an input sequence*x*, so the training standards can be defined as follows:

(3) $$\begin{array}{c} \underset{\theta }{\max}\frac{1}{K}\overset{k}{\underset{k=1}{\sum }}\log\left(\;y_{k}\;|\;x_{k}\;\right) \end{array}$$

where *θ* is a collection of network parameters and *k* designates the size of the training set. As mentioned before, recurrent functions in encoder-decoder models are not usual mathematical functions. RNNs are not powerful enough to capture all features about sequences, so more powerful choices, such as LSTM RNNs are required.

## 5. Proposed Multitask Learning Model for Arabic Dialects NMT

The emergence of deep learning approaches such as RNN or CNN models as discussed by Matthieu et al. [23] are considered as reasonable methods which are applicable throughout different natural language processing tasks. Also, several new approaches were observed to combine several related tasks in one unified model such as multitask learning (MTL). MTL is an approach to inductive transfer that improves generalization by using the domain information contained in the training signals of related tasks as an inductive bias. Given *m* learning tasks {*T*_*i*_}_*i*=1_^  *m*^ where all the tasks or a subset of them are related but not identical, MTL aims to help improve the learning of a model for *T*_*i*_ by using the knowledge contained in the *m* task. MTL utilizes the correlation and the shared representation between related translation tasks such as MSA-ENG and AD-MSA to improve translation quality by learning tasks in parallel. What is learned for each task can help other tasks be learned better. The goal of inductive transfer is to leverage additional sources of information to improve the performance of learning on the current task. Inductive transfer can be used to improve generalization accuracy, the speed of learning, and the intelligibility of learned models. A learner that learns many related tasks at the same time can use these tasks as inductive bias for each other and thus better learn the domain's regularities. This can make learning more accurate and may allow hard tasks with small amount of training data to be learned.

MTL allows features developed in the hidden layer for one task to be used by other tasks. In addition, it also allows features to be developed to support several tasks that would not have been developed in any single task learning (STL) net trained on the tasks in isolation. Importantly, MTL also allows some hidden units to become specialized for just one or a few tasks. Other tasks can ignore hidden units they do not find useful by keeping the weights connected to them small. MTL is achieved by adhering to hard or soft parameters and through the sharing of the hidden layers. Hard parameter sharing is applied by sharing the hidden layers of across all tasks, since it reduces the risk of overfitting. In soft parameter sharing, each translation task has its own model and particular parameters.

This research established one single unified MTL model to do translations for all the language pairs rather than training a language pair with high resource (parent model) and then to transfer the parameters that were learnt in the parent model to language pair with low resource (child model) for initialization and training. The overall model automatically has the ability to learn and share the required knowledge and information between all the necessary translation tasks. The proposed model is utilized for the translation of two source languages such as AD and MSA and two targets languages which are MSA and English, respectively. The architecture of the model designed in this section is a recurrent neural network (RNN) based on encoder-decoder architecture with two target tasks, and every individual task is a particular translation direction. All translation tasks share one translation decoder across all the various language pairs such as MSA-ENG and AD-MSA. Sharing more information across the tasks is preferred, and the model details are described in this section. Also, the training schedule for each individual task will be discussed.

### 5.1. Model Architecture

**Figure 2:**
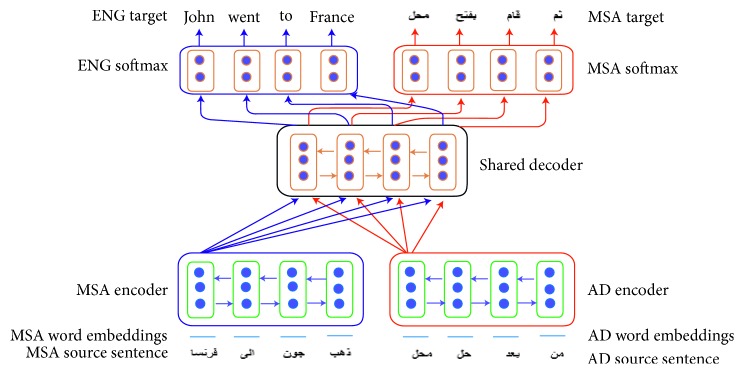
The architecture of the proposed model.

The general architecture of the encoder-decoder model has two parts: The encoder E and the decoder D.  Figure 2 gives a brief description and a summary of this architecture. The baseline considers this scenario where one model is used for all the tasks related to the translation. The first task is for translation from Modern Standard Arabic (MSA) to English and the second task for translation from Arabic dialect (AD) to MSA. Therefore; all parts (two encoders and one shared decoder) stand for all tasks. We will have three components, E1_MSA and E2_AD components as two and D_ENG, D_MSA as one, in total. One of the main decisions for designing multitask learning architecture is the level of sharing across the tasks which is very beneficial in translating Arabic dialects to MSA. It was motivated by the recommended machine translation architecture for multiple languages [24–26], and the impact of sharing one translation decoder in the output quality of the model has been analysed. Sharing more parameters across the translation tasks (MSA-English and AD-MSA) by using the shared decoder, the model will be suitable enough to learn more from the training set and able to capture more morphological, semantic, lexical, and syntactic features for the Arabic dialects and give a better the performance of the AD-MSA translation task where enough Arabic dialect data are not available, and it is considered as a low-resource language.

The sharing of decoder hidden layers (LSTM layers followed by a dropout layer and two dense layers) between the translation tasks is useful particularly for the low-resource language pairs. In MTL framework, the amount of source language is not limited by the low-resource language pairs, and the proposed model is able to learn better representation for the source language. The representation of the source language learned from the multitask model is more stable and can be viewed as a constraint that improves the translation quality for the Arabic dialects. Therefore, the data rareness problem and the overfitting problem can be alleviated for the language pairs with only small amount of the training data. MTL improves generalization by leveraging the domain-specific information contained in the training signals of related tasks. It doses this by training translation tasks for AD-MSA and MSA-ENG in parallel while using a shared representation. In effect, the training signals for the extra task serve as an inductive bias.

### 5.2. Encoder Side

In this architecture, two bidirectional long short-term memories (Bi-LSTMs) are used as encoders for all tasks, one Bi-LSTM encodes the Arabic dialect (AD) sentence and another Bi-LSTM encodes Modern Standard Arabic (MSA) sentence. As mentioned before, it is seen that normal mathematical functions in the encoder-decoder-based architectures does not consider recurrent functions. Also, the conventional recurrent neural network (RNN) is not adequately capable to obtain and capture all knowledge about the sequences, so more powerful and robust alternatives such as Bi-LSTM RNNs are needed. LSTM units alleviate the issue of long-distance dependencies by boosting the RNN with a memory vector *m*_*t*_ ∈ *R*^*d*^. An LSTM unit takes*x*_*t*_, *h*_*t*−1_, and *m*_*t*−1_ as its input and produce *h*_*t*_ and *m*_*t*_ by computing the following equations:

(4) it=σWixt+Uiht−1+bi,ft=σWfxt+Ufht−1+bf,ot=σWoxt+Uoht−1+bo,gt=tanh⁡Wgxt+Ught−1+bg,mt=ft⊙mt−1+it⊙gtht=ot⊙tanh⁡mt

where *i*_*t*_, *f*_*t*_, and *o*_*t*_ designate the input, forget, and output gates, respectively. These gates collectively determine how to update the current memory cell *m*_*t*_ and the current hidden state *h*_*t*_. The parameter *d* is used to indicate the memory dimension in the LSTM where all vectors in the defined architecture have the same dimension. *σ*(.) is an element-wise sigmoid function with an output range between [0,1]. Subsequently, tanh indicates the hyperbolic tangent function that has an output range between [−1,1]and ⊙ denotes the element-wise multiplication function and *W*_*p*_, *U*_*p*_ and *b*_*p*_, *p* ∈ {*i*, *f*, *o*, *g*} are considered as the network parameters. The function *f*_*t*_ is set to have a better understanding of mechanisms involved in the architecture and to control distinct type of information that is needed to be discarded from the old memory cell. In addition, the use of *i*_*t*_ to control the amount of information that is stored in the current memory cell and *o*_*t*_ to control the parameters is required to be provided as an output based on the memory cell *m*_*t*_.LSTMs are designed to learn long-term dependencies of time-series data.

**Figure 3:**
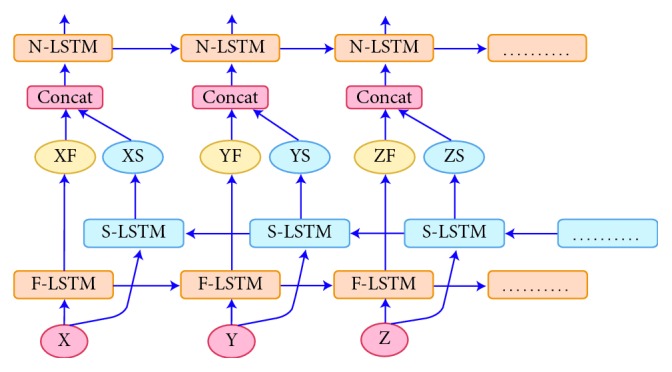
The layout of the bidirectional LSTM encoder.

In neural machine translation systems, it is necessary to translate the required knowledge to specific words such that the target language could arise in the source language. The source-side knowledge is generally found to be read from left-to-right, similar to the target side as in European languages and other Asian languages. The source-side information can also be represented from right-to-left, similar to the target side as in the Arabic language. Therefore, by considering the language pair, information regarding a specific output word is spread and split up into specific ranges of the input side. This procedure was performed to achieve the best possible context at each point in the encoder network; from this research, it can be seen to use a bidirectional RNN [27] as an encoder.  Figure 3 shows the design of bidirectional LSTMs. The first LSTM (F-LSTM) layer read the source sentence from left-to-right, while the second LSTM(S-LSTM) layer read the same source sentence from right-to-left. The outputs from F-LSTM and S-LSTM are first concatenated and then fed to the next layer (N-LSTM). This process occurs for all translation tasks in all encoders that use Bi-LSTM.

### 5.3. Decoder Side

One shared decoder is used for all the translation tasks. This research work explores if it is reasonable to share all the information across the translation tasks between all language pairs and let the model learn how to represent these tasks. Therefore, in this design, one decoder is shared. The decoder has several common hidden LSTM layers followed by the dropout layer and two dense layers. These dense layers were activated by the ReLU activation function. The shared decoder will model the generation of the target words for both English and MSA. Therefore, we have two encoders E1_MSA, E2_AD, and one shared decoder D_ENG, D_MSA. With one shared decoder, we used two output layers for the translation tasks. Each output layer is composed of the softmax layer.  Figure 2 describes the shared layers depending on the architecture of the model. In the proposed multitask learning model, hard parameter sharing was performed by sharing the common hidden layer within the decoder function across the related tasks.

### 5.4. Task-Specific Output Layer

In a single specific task, a simple method is to map the input sequence to a fixed-sized vector by using one Bi-LSTM encoder and then to feed the vector to the shared Bi-LSTM decoder and then to the softmax layer for the translation task. Given a text sequence = {*x*_1_, *x*_2_,…, *x*_*T*_}, first a lookup layer was used to get the vector representation (embeddings) *x*_*i*_ of each word **x**_**i**_. The output at the Bi-LSTM decoder can be regarded as the representation of the whole sequence. The output of the decoder is fed to a softmax nonlinear layer that predicts the probability distribution over output vocabulary.

### 5.5. Optimization

**Figure 4:**
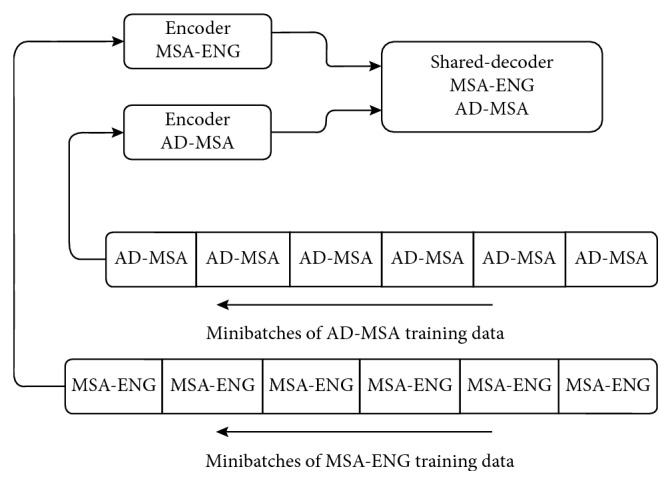
The layout of the optimization technique.

The optimization method used is Adam which is considered as an efficient algorithm for gradient-based optimization of stochastic objective function [28]. Many minibatches were learned with fixed sizes within a language pair (MSA-English) for number of iterations and then continue on the next language pair (AD-MSA). The layout of our optimization method is shown in Figure 4.

### 5.6. Training Schedule

Minibatches of size 256 tokens were used throughout the experiments. The weight updates are set by using the Adam optimization algorithm. The main idea involved in the research is the introduction of training samples into the optimization algorithm that will be used for training. We take into consideration of one task in each minibatch. Also, we have the same model architecture and parameters settings for the whole translation tasks. The model has different weights for the individual tasks due to a default training schedule. Initially, the model training is performed on the MSA-ENG translation task and then training on the AD-MSA translation task. This process was continued alternately. The significant improvement to be conducted in the proposed work is the AD-MSA translation task.

## 6. Results and Discussion

Neural machine translations experiments were presented by using the multitask learning approach on different translation tasks: machine translations from Modern Standard Arabic (MSA) to English and translation from Arabic dialects (AD) to Modern Standard Arabic (MSA). The experiments were conducted on two types of Arabic dialects: Levantine and Maghrebi. Levantine Arabic is a spoken dialect in Syria, Jordan, Palestine, and Lebanon. Maghrebi Arabic is spoken variety of Standard Arabic widely used in Morocco, Algeria, and Tunisia. Neural machine translation models based on the multitask learning approach will be used for sparse data. Further, 10000 pair parallel corpus were deployed for the MSA-ENG translation task.

### 6.1. Data

We concatenated the Levantine dialects (Jordanian dialect, Syrian dialect, and Palestinian dialect) together from PADIC and from MPCA Corpus as well we concatenated the Maghrebi dialects (Moroccan dialect, Algerian dialect, and Tunisian dialect) together from the same corpuses. Consequently, 13805 sentence pairs were trained for Levantine dialects (LD) and 17736 sentence pairs for Maghrebi dialects (MD) that are collected from TV shows, movies, and social media. For the test set, we used 2000 sentence pairs for Levantine dialects and 2000 sentence pairs for Maghrebi dialects. The diacritics, punctuations, and non-Arabic characters were removed during the preprocessing stage for Arabic dialects and MSA. Besides, Arabic tokens are separated by whitespace except in instances of quoting English-style abbreviations and are tokenized Arabic dialects (Levantine and Maghrebi), Modern Standard Arabic, and English languages using python tokenizer with the default settings for English. Also, orthographic normalization was performed. For instance, transformation of all إأٱآ characters to ا character was done. No stemming or stop word removal has been done. The sequence length is set to 55. The MSA contains a wider variety of tokens than English and Arabic dialects, and its sentences are shorter than English and Arabic dialects. Modern Standard Arabic has more probability in its infrequent words than English. The words in the long tail tend to be morphologically complex, alternately beginning with affixes like “AL” “ال” (the) or “wa” “و” (and).

### 6.2. Training

We use 19,327-word vocabularies for Levantine Arabic (LA) and 22,459-word vocabularies Maghrebi Arabic (MA) on the AD-MSA translation task. In addition, 10,185-word vocabularies for Modern Standard Arabic are used on the MSA-ENG translation task. The digits were not normalized. Adam optimization is used with *β*1 = 0.9 and *β*2 = 0.999, a vertical dropout of 0.5, and gradient clipping beyond an absolute value of 5. The training is performed on GPU in batches of 256 randomly selected training pairs until the training loss begins to increase, annealing an initial learning rate of 0.001. The initial learning rate is actively selected as the largest learning rate that led to good activations and large but possible updates. The proposed model incorporates (3M trainable parameters for the MSA-ENG task, 6M trainable parameters for the LA-MSA task, and 8.85M trainable parameters for the MA-MSA task). Word embedding and hidden size are shown in Table 1. This model size proved to require 71 seconds per epoch for the LA-MSA task, 102 seconds per epoch for the MA-MSA task and 502 seconds per epoch for the MSA-ENG task. The model was trained alternately on MSA-ENG and AD-MSA tasks. The training data will be randomly shuffled at each epoch for all tasks. For two parallel sentence pair corpora, the model is trained by minimizing the cross-entropy loss for each translation task.

### 6.3. Experimental Results

The results obtained were summarized to demonstrate the efficiency of our proposed multitask learning model. The multitask learning model is trained concurrently on all three training datasets and compared with the BLEU scores with models that were trained individually on each dataset. Table 1 shows the BLEU scores on the test dataset. Models which learned from the multitask learning structure outperform the models trained independently. The results in Table 1 show that translation performance on all the target languages was enhanced due to the given small dataset of the Levantine Arabic-MSA. This result makes sense because of the closeness of the Levantine dialect to the Modern Standard Arabic (MSA), and related languages serve each other by sharing the same vocabularies. Also, it was noticed that the improvement in terms of the BLEU score was as a result of data amplification. Data amplification which is an effective increase in sample size due to extra information in the training signals of related tasks. Amplification occurs when there is noise in the training signals. Consider two tasks, B and C, with independent noise added to their training signals, that both benefit from computing a hidden layer feature F of the inputs. A net learning for both B and C can use the two training signals to learn F better by averaging F through the noise. This happens if the net learning recognizes that the two tasks share F. The simplest case is when B=C. Furthermore, it was observed that Magribi Arabic is from the Arabic dialects and was derived from different substrata and a mixture of many languages Berber, Latin (African Romance), old Arabic, Turkish, French, Spanish, Mozarabic, Italian, and Niger-Congo languages, integrating new English and French words. The proposed multitask learning model was able to improve the translation performance on the training dataset of Magribi Arabic-MSA and demonstrate the generalization of our proposed model to multiple-target languages such as the MSA-ENG translation task.

### 6.4. Model Analysis and Discussion

The experiments were done to explain why the multitask learning model works better than the model trained independently on the multiple-target machine translation. The speed of model convergence for multitask learning is quicker than models trained individually, consequently when a model is trained for the resource-poor language pair. Also, the sharing decoder parameter is found to be useful for the resource-poor languages. The amount of the source language in multitask learning models is not restricted by the resource-poor language pairs and is capable of learning a better representation of the source language. Furthermore, the representation of the source language which is learned from the multitask model is steadier and can be seen as a constraint that leverages translation performance of all language pairs. Therefore, with a few training examples the problem of overfitting and data scarcity will be eased for language pairs. The multitask learning model produces translations with high quality. Few examples are shown in Tables 2–4. The examples are from the test dataset. The MSA translations generated by the proposed multitask learning model and the single model for the Levantine Arabic and Magribi Arabic are shown in the table. One of the common problems of many neural machine translation systems is that they do not translate some specific parts of the source sentence or that parts of the source sentence are translated two times. As shown in the first two examples in table, the baseline model or single model did not translate many parts of the source sentence or gave wrong translations. The translation performance has improved significantly with multitask learning approach. In the first example, the single model or baseline model is not translating the words وانتو to انتم and خلصتوا to انهيتم and العزايم to الدعوات while the Multitask learning model translated correctly. Also, the multitask learning model dose not generate the exact translation that matches the reference. As shown in the third example for Magribi Arabic, the multitask model did not generate the word يا and repeated the word زينب twice. The MTL model achieved perfect translation performance on different languages as shown in Table 3 for the MSA-ENG translation task. The multitask learning model in general is able to generate correct sequence and able to translate Arabic dialects sentences and convey information about verb, subject, and object. Furthermore, the proposed MTL model can handle free word order issue in Arabic dialects.

## 7. Conclusions

In this work, the challenges observed in the translation of Arabic dialects to the Modern Standard Arabic (MSA) were studied. Furthermore, the proposed research developed a multitask learning model based on the recently proposed recurrent neural network-based encoder-decoder architecture.

In this research, a unified neural machine translation model was trained in which the decoder is shared over all language pairs, and each source language has a separate encoder; this due to the reason that each Arabic dialect has its own peculiarities and orthography. As far as we know, neural machine translation models from Arabic dialect to modern standard form have not been investigated. Experiments demonstrate that given a small parallel training data, the multitask neural machine translation model is effective to generate the correct sequence, produce translations of high quality, and learn the predictive structure of multiple targets. Moreover, our proposed multitask learning model is able to address the problem of data scarcity and the problem of the insufficiency of the slandered orthographies for Arabic dialects. Our proposed neural machine translation model is practical and efficient and is found to provide faster and better convergence for both low-resource languages and rich resource languages under the multitask learning framework. In this paper, the performance of the machine translation task was improved by using the multitask learning approach. In the future, we will continue our work in more practical settings. For example, we will investigate the performance of the model using attention approach.

## Figures and Tables

**Table 1:** Multitask neural translation vs single model.

Model	Pairs	Embedding size	Hidden size	Epochs	Blue
Single NMT	LA-MSA	150	170	120	0.17
Multitask	LA-MSA	150	170	170	**0.41**
Single NMT	MA-MSA	180	180	120	0.16
Multitask	MA-MSA	180	180	230	**0.30**
Single NMT	MSA-EN	160	160	120	0.10
Multitask	MSA-EN	150	170	170	**0.27**

**Table 2:** Translation examples for Levantine Arabic.

Levantine Arabic	اه و انتو خلصتوا العزايم
Reference MSA	نعم و انتم انهيتم الدعوات
Single MSA	نعم صحيح في فريضة تدرسه
Multitask MSA	نعم و لكن انهيتم الدعوات

**Table 3:** Translation examples for Magribi Arabic.

Magribi Arabic	تيقيني يا زينب معارفش
Reference MSA	صدقيني لا اعلم يا زينب
Single MSA	صدقيني مثلما حالكم؟ زينب
Multitask MSA	صدقيني لا اعلم زينب زينب

**Table 4:** Translation examples for MSA.

MSA Arabic	هناك برتقالة على الطاولة
Reference ENG	There is an orange on the table
Single ENG	There is in the table
Multitask ENG	There's are orange on the table floor

## Data Availability

The datasets generated during the current study are available in [AD_NMT] repository (https://github.com/laith85/AD_NMT).
